# Bioinspired Molecular Magnetic Field-Responsive Catalyst for On-Demand Switching of 2e^−^ and 4e^−^ Oxygen Reduction

**DOI:** 10.1007/s40820-026-02319-0

**Published:** 2026-08-10

**Authors:** Boying Zhang, Qiaoling Guo, Haochuan Li, Yue Wang, Qing Li, Haining Liu, Shanlin Qiao

**Affiliations:** 1 College of Chemistry and Pharmaceutical Engineering, Hebei University of Science and Technology, Shijiazhuang, 050018 People’s Republic of China; 2 Hebei Research Center of Pharmaceutical and Chemical Engineering, Shijiazhuang, 050018 People’s Republic of China; 3 Hebei Engineering Research Center of Organic Solid Photoelectric Materials for Electronic Information, Shijiazhuang, 050018 People’s Republic of China

**Keywords:** Molecular magnetic field-responsive catalyst, Metal-Salen covalent organic frameworks, Oxygen reduction reaction, Selectivity regulation

## Abstract

Achieving precise and on-demand steering of the oxygen reduction reaction (ORR) pathway between the efficient 4e^−^ route to H_2_O and the valuable 2e^−^ route to H_2_O_2_ remains a pivotal challenge in electrocatalysis. Herein, we address this challenge by designing a bioinspired molecular magnetic field-responsive catalyst (MMFR-C) via magnetic single-atom-anchored Salen-based covalent organic frameworks (MSA-Salen COFs) onto magnetic nanoparticles (single/multi-domain Fe_3_O_4_). Mimicking cytochrome *c* oxidase, the MMFR-C employs MSA-Salen COFs as an ordered proton-transfer channel and well-defined N_2_-M-O_2_ moieties as enzymatic O_2_ activation sites, with Fe_3_O_4_ providing a built-in magnetic field for remote regulation of the active-site electronic structure. The bioinspired MMFR-C exhibits switchable ORR pathways. Relative to the pristine Co-Salen COF (26% H_2_O_2_ selectivity, *n* = 3.48), the MMFR-C integrated with a single-domain Fe_3_O_4_ exhibits a remarkably enhanced H_2_O_2_ selectivity of 63.9% (*n* = 2.72), while that with a multi-domain Fe_3_O_4_ diverts the ORR pathway toward the 4e^−^ route (*n* = 3.67). (i) We elucidate that the uniform magnetic field from the single-domain Fe_3_O_4_ in MMFR-C favors orbital hybridization between its active N_2_-M-O_2_ moieties and the *OOH intermediate, with moderate *OOH adsorption suppressing O–O scission and thus steering ORR selectivity toward H_2_O_2_. (ii) In contrast, the enhanced specific magnetism from its multi-domain Fe_3_O_4_ core optimizes the *d*-band center of MMFR-C’s active sites, stabilizes triplet O_2_ adsorption, and reduces spin-forbidden transition barriers, thereby facilitating O–O cleavage and diverting its ORR pathway to the 4e^−^ route.

**Supplementary Information:**

The online version contains supplementary material available at 10.1007/s40820-026-02319-0.

## Highlights

- The bioinspired molecular magnetic field responsive catalysts were synthesized to selectively switch between the 2e^−^ and 4e^−^ oxygen reduction reaction (ORR) pathways.
- The molecular magnetic field responsive catalyst integrated with single domain Fe_3_O_4_ delivers a significantly boosted H_2_O_2_ selectivity of 63.9% (electron transfer number, *n* = 2.72), whereas the multi domain Fe_3_O_4_ incorporated variant effectively redirects the ORR exclusively toward the 4e^−^ pathw ay (*n* = 3.67).

**Supplementary Information:**

The online version contains supplementary material available at 10.1007/s40820-026-02319-0.

## Graphical abstract

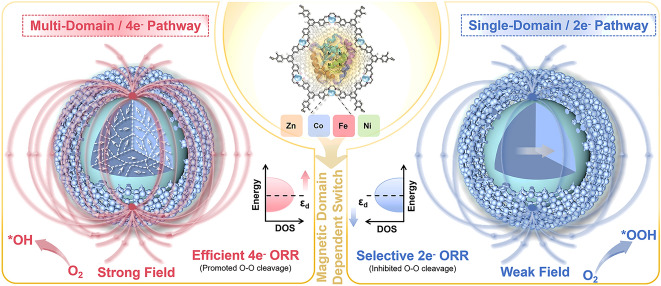

## Introduction

The highly efficient oxygen reduction reaction (ORR) is found in nature, which plays a pivotal role in biological respiration and electrochemical energy conversion [1, 2 ]. However, achieving precise and on-demand steering of the ORR pathway between the efficient 4e^−^ ORR pathway to H_2_O and the valuable 2e^−^ ORR to H_2_O_2_ remains a pivotal challenge [3–6]. This difficulty originates from the complex multi-electron/proton-coupled kinetics of ORR, where the critical bifurcation is dictated by whether the oxygen–oxygen (O–O) bond cleaves [7–9]. Over the past years, strategies for controlling O–O bond cleavage strategies, including metal atom doping [10–13], heterojunction engineering [14, 15 ], and coordination environment optimization [16–19], have primarily relied on structural modifications of the active sites. However, such approaches inherently require alteration of the catalyst structure. In contrast, applying an external magnetic field provides a non-invasive and sustainable means to tune both activity and selectivity in real time, without altering the catalyst itself [20–22]. A recent study demonstrates the application of the magnetic modulation of the *eg*-orbital occupancy of Co ions to boost the two-electron oxygen reduction electrocatalysis for faster water decontamination [23]. Moreover, the built-in magnetic field can transfer the Co center from a low-spin (LS) state to a high-spin (HS) state without changing the atomic structure, thereby increasing the turnover frequency by an order of magnitude, enhancing the H_2_O_2_ selectivity of the ORR by 50% [24]. Prior efforts have mainly focused on magnetic field-enhanced electrocatalytic activity, yet the rational switching of ORR selectivity on the foundation of improved activity remains highly desirable and largely underexplored.

In nature, cytochrome *c* oxidase (CcO) catalyzes the reduction of oxygen to water, an essential reaction in cellular respiration [25]. In CcO, O_2_ binding and activation occur at the heme/Cu binuclear center. In the fully reduced state, the Cu^+^ species donates electrons to the adsorbed O_2_, facilitating the formation of the key Fe^3+^-peroxo-Cu^2+^ intermediate and subsequent cleavage of the O–O bond. This electron-donating effect provides the essential thermodynamic driving force for the complete 4e^−^ ORR pathway, leading to the exclusive production of H_2_O [26–28]. If the Cu center is locked in the Cu^2+^ state and loses its electron-donating capability, the electron density at the active site becomes insufficient to drive O–O bond scission. The peroxo intermediate thus remains stable with an intact O–O moiety, forcing the reaction to shift toward the 2e^−^ ORR pathway that generates H_2_O_2_ instead. Inspired by the dynamic electron-donating modulation via Cu valence evolution in CcO, where reversible electron transfer dictates ORR pathway selectivity. Molecular magnetic field can dynamically tune the electronic configuration, spin state, and electron-donating capability of metal sites. We reason that a molecular magnetic field could serve as a non-invasive and controllable tool to mimic this biological electron-regulating behavior in artificial catalytic systems. On this basis, we put forward the concept of constructing a bioinspired molecular magnetic field-responsive catalyst (MMFR-C), intended to regulate the electron-donation behavior of active metal sites and thereby achieve programmable switching between the 2e^−^ and 4e^−^ ORR pathways.

**Scheme 1:**
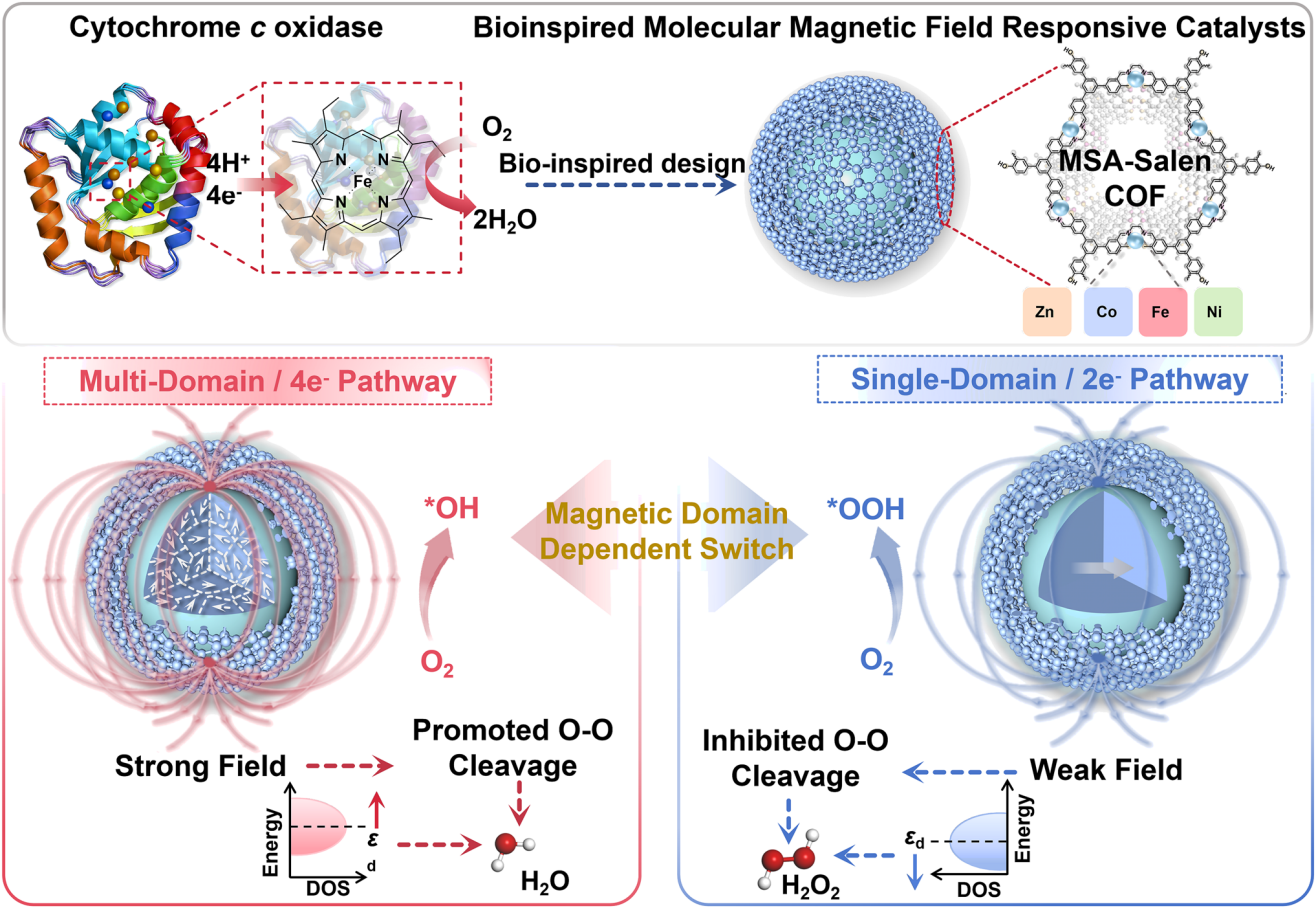
Schematic diagram of the design and catalytic mechanism of the MMFR-C catalyst

Herein, we materialize this concept by fabricating a class of bioinspired MMFR-C, achieved by anchoring magnetic single atoms in Salen-based covalent organic frameworks (Salen COFs) (Metal = Zn, Fe, Co, Ni) that parcel magnetic Fe_3_O_4_ nanoparticles (Scheme 1). As a cytochrome *c* oxidase mimic, MMFR-C integrates ordered proton-transfer channels from magnetic single-atom-anchored (MSA)-Salen COFs and well-defined N_2_-M-O_2_ moieties as efficient O_2_ activation sites, while the embedded Fe_3_O_4_ component generates a built-in magnetic field that remotely modulates the electronic structure of the active sites. Notably, the bioinspired MMFR-C enables controllable switching between ORR pathways simply by varying the magnetic domain structure of Fe_3_O_4_. Specifically, MMFR-C incorporating single-domain Fe_3_O_4_ delivers high H_2_O_2_ selectivity (63.9%, *n* = 2.72), whereas the counterpart with multi-domain Fe_3_O_4_ redirects the reaction toward the 4e^−^ pathway (*n* = 3.67), both exhibiting superior tunability relative to the pristine Co-Salen COF (H_2_O_2_ selectivity = 26%, *n* = 3.48). It is further confirmed that the weak, uniform magnetic field in single-domain Fe_3_O_4_ gives rise to moderate *OOH adsorption that inhibits O–O cleavage, favoring the 2e^−^ ORR toward H_2_O_2_. By contrast, the stronger intrinsic field in multi-domain Fe_3_O_4_ optimizes the *d*-band center and lowers spin-transition barriers, facilitating O–O cleavage and promoting the 4e^−^ route.

## Experimental

### Materials

Reagents and solvents, readily available commercially, were utilized without additional purification. Detailed information is available in the Supporting Information.

### Synthesis of Zn-Salen COF

1,3,5-tris(4′-hydroxy-5′-formylphenyl)benzene (THB) (0.03 mmol, 13.2 mg) and Zn(OAC)_2_·2H_2_O (14.8 mg) were weighed into a Pyrex tube (volume of ca. 10 mL). The mixture was dissolved in 1.5 mL of mesitylene/ethanol (1:1 v/v) and sonicated for 5 min. Then, ethanediamine (0.045 mmol, 5 μL) was added to the mixture. The mixed solution was ultrasonicated for another 2 min. After the aqueous acetic acid (6 M, 0.15 mL) was added, the solution was sonicated for 5 min to ensure uniform dispersion. The Pyrex tube was degassed through three freeze–pump–thaw cycles and then flame-sealed. The tube was placed in an oven at 120 °C for 3 days. When the reaction time was up, the ampoule was cooled to room temperature and opened. The product was collected by centrifugation and then cleaned with N, N-dimethylformamide (DMF), ethanol, and acetone. The powder was dried in an oven at 100 °C under vacuum overnight.

### Synthesis of MSA-Salen COFs

The Zn-Salen COF and M(OAc)_2_·nH_2_O were weighed into a glass vial, and dry ethyl alcohol was added. The mixture was continuously stirred at room temperature for 48 h, with the solution being refreshed three times. The solid was collected by centrifugation and washed with ethyl alcohol and acetone several times. The powder was dried in an oven at 80 °C under vacuum overnight (Metal: Fe, Co, Ni).

### Synthesis of s-Fe_3_O_4_@Zn-Salen COF

Weigh single-domain nanoparticles s-Fe_3_O_4_ (4.6 mg) and add it to approximately 10 mL of a Pyrex tube, then add a mesitylene/ethanol (1.5 mL, 1:1 v/v) solution and ultrasonically treat for 10 min to disperse the Fe_3_O_4_ homogeneously. Next, add ethylenediamine (0.045 mmol, 5 μL) and sonicate for 5 min. Subsequently, weigh THB (0.03 mmol, 13.2 mg) and Zn(OAc)_2_·2H_2_O (14.8 mg) and add them to the mixed solution, followed by ultrasonic treatment for 10 min. After adding aqueous acetic acid (6 M, 0.15 mL), continue ultrasonic treatment for 5 min to ensure uniform dispersion. Degas the Pyrex tube via three freeze–pump–thaw cycles and seal it by flame. Place the tube in an oven at 120 °C and dry for 3 days. At the end of the reaction, cool the ampoule to room temperature and open it. Collect the product by centrifugation and wash with DMF, ethanol, and acetone. Dry the powder overnight in a vacuum oven at 100 °C to obtain s-Fe_3_O_4_@Zn-Salen-COF (20 wt% Fe_3_O_4_) composites of various sizes.

### Synthesis of m-Fe_3_O_4_@Zn-Salen COFs

The composite m-Fe_3_O_4_@Zn-Salen COF (20 wt% Fe_3_O_4_) was prepared using a procedure identical to that described for s-Fe_3_O_4_@Zn-Salen COF, except that multi-domain Fe_3_O_4_ nanoparticles (m-Fe_3_O_4_, ≈150 nm) were used in place of the single-domain nanoparticles (s-Fe_3_O_4_, ≈10 nm). All other reactants, solvents, and reaction conditions remained the same.

### Synthesis of s/m-Fe_3_O_4_@MSA-Salen COFs

The s/m-Fe_3_O_4_@Zn-Salen COFs and M(OAc)_2_·nH_2_O were weighed into a glass vial, and dry ethyl alcohol was added. The mixture was continuously stirred at room temperature for 48 h, with the solution being refreshed three times. The solid was collected by centrifugation and washed with ethyl alcohol and acetone several times. The powder was dried in an oven at 80 °C under vacuum overnight (Metal: Fe, Co, Ni).

### Electrochemical Performance Tests

Electrochemical measurements were performed using a rotating ring-disk electrode (RRDE) system integrated with a CHI 760E electrochemical workstation. A glassy carbon disk/platinum ring RRDE electrode (Ø = 5.61 mm) from PINE was used as the work electrode. A graphite rod and an Ag/AgCl electrode were used as the counter and reference electrodes, respectively. All potentials were converted to the reversible hydrogen electrode (RHE) scale using the Nernst equation (Eq. 1).

1 $$E_{{{\mathrm{RHE}}}} = E_{{\mathrm{Ag/AgCI}}} + 0.197 + 0.0591 \times pH$$

Each catalyst sample (5 mg) was dispersed in 1 mL of a mixed solution comprising ethanol, deionized water, and Nafion solution (5 wt%) in a volume ratio of 9:10:1 (v/v/v). The resulting suspension was sonicated for 30 min to ensure homogeneous dispersion. 10 μL of ink was loaded onto the RRDE electrode and dried at 500 rpm. Before testing, the electrolyte was saturated with oxygen by continuously passing oxygen through the 0.1 M KOH electrolyte for 30 min. Subsequently, a 30-cycle cyclic voltammetry (CV) sweep (sweep rate 50 mV s^−1^) was performed to activate the catalyst, with the oxygen continuously applied during the test. The catalyst was stabilized when the CV curve remained essentially unchanged. After the CV activation was completed, the test was suspended again, and oxygen was added for 20 min to replenish the dissolved oxygen consumed during the CV test, and then, the LSV test was performed. A linear voltage of 0.2‒0.8 V vs. Ag/AgCl was applied to the disk electrode, and 1.3 V vs. RHE was applied to the ring electrode, with a scan rate of 10 mV s^−1^ and a RRDE rotation speed at 400‒1600 rpm. After 30 min of continuous nitrogen in the electrolyte, a CV scan was performed to test the background current in this system to verify that the reaction occurring in the system was an oxygen reduction reaction.

## Results and Discussion

### Synthesis and Characterization

The bioinspired MMFR-C (s-Fe_3_O_4_@Zn-Salen COF and m-Fe_3_O_4_@Zn-Salen COF) was synthesized according to an in situ growth strategy, using THB, Zn(OAC)_2_·2H_2_O, and ethane diamine in the presence of single-/multi-domain Fe_3_O_4_ with mesitylene and ethanol as the solvent, as well as acetic acid aqueous solution as the catalyst. Zn-Salen COF can be synthesized under the above conditions in the absence of Fe_3_O_4_ according to the previous method [29]. The framework features readily accessible 1D channel arrays and dynamic N_2_-Zn-O_2_ coordination bonds, which facilitate metal ion exchange [30]. Therefore, the as-synthesized Zn-Salen COF, s-Fe_3_O_4_@Zn-Salen COF, and m-Fe_3_O_4_@Zn-Salen COF were immersed in saturated aqueous solutions of metal acetate salts, including Co(OAc)_2_·2H_2_O, Fe(OAc)_2_·H_2_O, and Ni(OAc)_2_·4H_2_O for 2 days. Inductively coupled plasma optical emission spectrometry (ICP-OES) was used to analyze the metal content for Ni-Salen COF, Co-Salen COF, and Fe-Salen COF (Table S1). The Ni, Co, and Fe contents in the Ni-Salen COF, Co-Salen COF, and Fe-Salen COF are 5.48, 5.65, and 5.22 wt%, respectively.

**Fig. 1:**
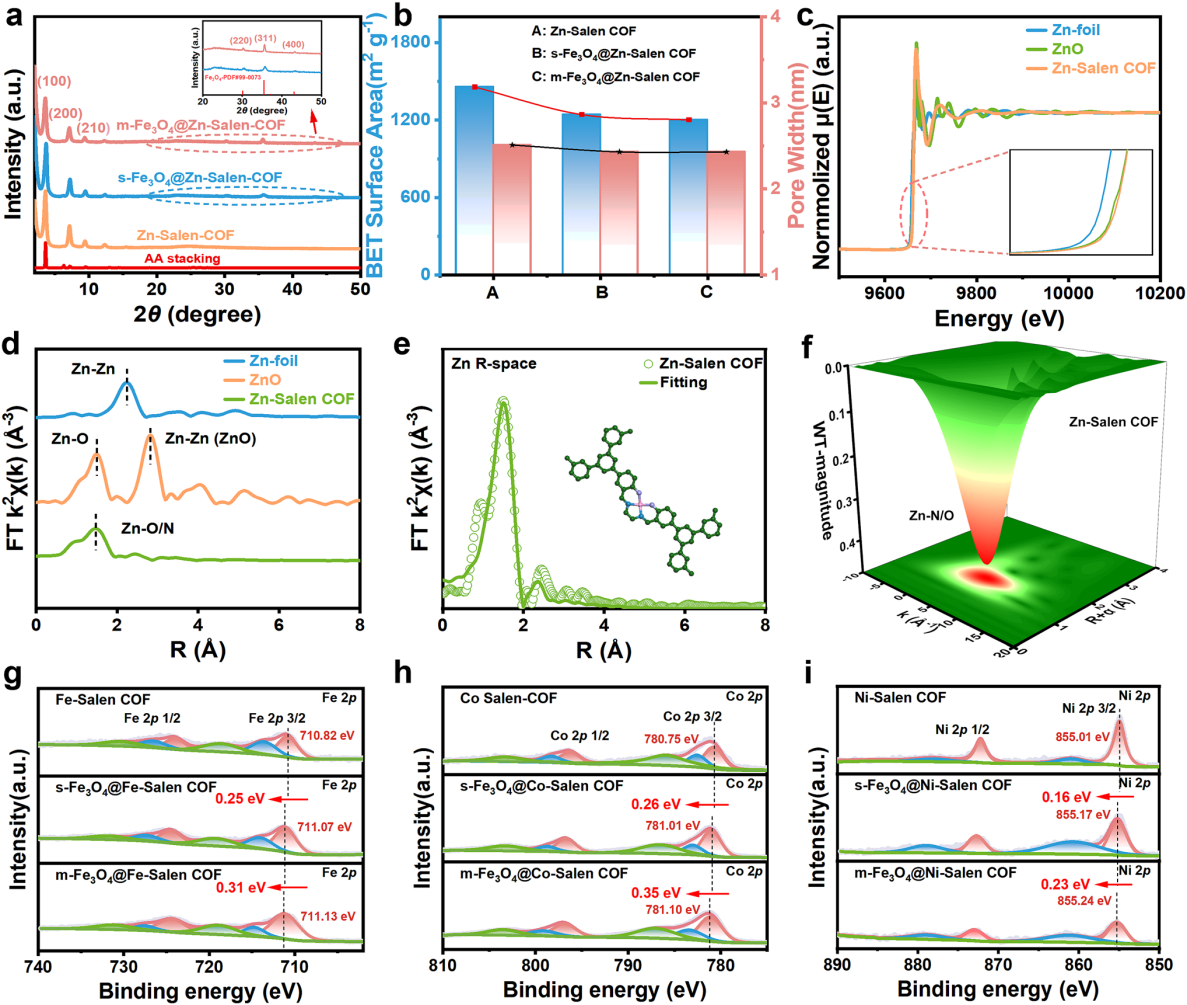
**a** PXRD pattern and **b** Brunauer–Emmett–Teller (BET) surface area, and total pore volume of Zn-Salen-COF and s/m-Fe_3_O_4_@Zn-Salen-COF, **c** Zn K-edge XANES spectra for Zn-foil, ZnO, and Zn-Salen-COF. **d** Fitting result of the EXAFS spectra of Zn-Salen COF at R space. **e** Corresponding *k*^2^-weight Fourier transforms of the Zn K-edge EXAFS spectra. **f** WT of the Zn *k*-edge of 3*d* for Zn-Salen COF. High-resolution XPS spectra of **g** Ni 2*p*, **h** Co 2*p*, and **i** Fe 2*p* of MSA-Salen COFs and s-Fe_3_O_4_@MSA-Salen COFs, respectively

Fourier transform infrared spectroscopy (FT-IR) spectra of THB, MSA-Salen COF, and MMFR-C (Fig. S2) reveal the disappearance of the characteristic C=O stretching vibration at 1655 cm^−1^, while peaks corresponding to C=N bonds emerge at 1630 and 1134 cm^−1^. This confirms the successful polycondensation between THB and ethane diamine. As shown in Fig. 1a, the powder X-ray diffraction (PXRD) patterns of Zn-Salen COF, s-Fe_3_O_4_@Zn-Salen COF, and m-Fe_3_O_4_@Zn-Salen COF exhibit strong diffraction peaks at 3.54°, 7.12°, 9.36°, and 12.32°, which correspond to the (100), (200), (210), and (220) facets, respectively. It is confirmed that the crystalline structure of the Zn-Salen COF is retained after the preparation of the MMFR-C.

These experimental diffraction features are consistent with the simulated pattern based on an AA-stacking model. Furthermore, the PXRD pattern of s/m-Fe_3_O_4_@MSA COF displays characteristic peaks at 30.3°, 35.7°, and 43.2°, which are indexed to the (220), (311), and (400) facets of Fe_3_O_4_ (JCPDS card no. 99–0073). As shown in Fig. S3a‒c, PXRD revealed that the MSA-Salen COF and MMFR-C retained the original crystal structure and high crystallinity after ion exchange.

The porosity of MSA-Salen COF and s/m-Fe_3_O_4_@Zn-Salen COF was studied by nitrogen sorption measurement at 77 K. As shown in Fig. S4a, b, the Zn-Salen COF and s/m-Fe_3_O_4_@Zn-Salen COF isotherms are type-IV isotherms with the characteristics of mesoporous, as indicated by the International Union of Pure and Applied Chemistry. The BET surface areas of Zn-Salen COF, s-Fe_3_O_4_@Zn-Salen COF, and m-Fe_3_O_4_@Zn-Salen COF were calculated to be 1456, 1242, and 1191 m^2^ g^−1^ (Fig. 1b). Pore size distribution was calculated by means of quenched solid density functional theory (QS-DFT). Notably, Zn‑Salen COF, s-Fe_3_O_4_@Zn-Salen COF, and m-Fe_3_O_4_@Zn-Salen COF share comparable pore sizes of 2.5 nm, suggesting that the introduction of Fe_3_O_4_ nanoparticles does not alter the intrinsic pore structure of the Zn‑Salen COF matrix (Figs. 1b and S4b).

Transmission electron microscopy (TEM) confirms the successful fabrication of MMFR-C. Spherical single-domain (s-Fe_3_O_4_, ~ 10 nm) and multi-domain (m-Fe_3_O_4_, ~ 150 nm) nanoparticles were employed (Figs. S5 and S6), and both are fully encapsulated by porous shells. High-resolution TEM (HRTEM) identifies a well-ordered 2.3 nm lattice fringe in the porous shell, which can be indexed to the (100) facet of Zn‑Salen COF, and a 0.29 nm lattice fringe characteristic of the (220) facet of spinel Fe_3_O_4_. Additionally, HRTEM confirms that metal-exchanged MMFR-C maintain well-defined morphologies and high crystallinity (Fig. S7a‒c).

To further investigate the local electronic environment and coordination geometry of Zn‑Salen COF, X‑ray absorption near‑edge structure (XANES) and extended X‑ray absorption fine structure (EXAFS) measurements were conducted at the Zn K‑edge. Zn K-edge XANES spectra of Zn-foil, ZnO, and Zn‑Salen COF (Fig. 1c) confirm that Zn atoms in Zn‑Salen COF are cationic, as their adsorption edges are higher than that of Zn foil. The Fourier-transformed EXAFS spectrum of Zn‑Salen COF (Fig. 1d) shows a single peak at 1.51 Å, assigned to the Zn–O/N scattering path [31], and the absence of a Zn‒Zn bond at ~ 2.2 Å indicates atomic dispersion of Zn. Fitting results of EXAFS in *R*, *k*, and *q* space (Figs. 1e and S8, Table S2) further confirm Zn atoms in Zn‑Salen COF adopt an N_2_-Zn-O_2_ coordination mode with two N and two O atoms, verifying the formation of a Zn–O/N coordination structure. Additionally, wavelet transform (WT) analysis (Fig. 1f) provides atomic-level resolution of metal distribution, with a single intensity maximum at ~ 5.0 Å^−1^ directly evidencing atomic dispersion of Zn ions in the N_2_-O_2_ chelating pockets of Salen ligands, forming well-defined N_2_-Zn-O_2_ single sites [32].

X-ray photoelectron spectroscopy (XPS) was used to determine the surface element concentration and chemical state. XPS spectra confirm the presence of C, N, O, and corresponding metal ions in both MSA-Salen COFs and s/m-Fe_3_O_4_@MSA-Salen COF, further verifying the successful incorporation of Ni, Co, and Fe atoms into Zn‑Salen COF and s/m-Fe_3_O_4_@Zn‑Salen COF structures. Notably, residual Zn^2+^ remains in Ni‑Salen, Co‑Salen, and Fe‑Salen COF after ion exchange, attributed to the kinetic equilibrium of coordination bonds. High-resolution N 1*s* and O 1*s* XPS spectra of MSA-Salen COFs and MMFR-C are displayed in Figs. S9‒S10. The deconvoluted N 1* s* spectra present two dominant peaks at 399.5 ± 0.2 and 400.5 ± 0.3 eV, corresponding to Metal−N and C=N bonds, respectively (Fig. S9). Similarly, the deconvoluted O 1* s* spectra show two peaks at 531.8 ± 0.6 and 532.5 ± 0.5 eV, assigned to O−Metal and C−O species, respectively (Fig. S10). This confirms that metal centers are anchored in N_2_-O_2_ ligands, consistent with EXAFS results. The slightly higher O–Metal content in MMFR-C than in pristine MSA-Salen COFs due to combined Fe–O (from Fe_3_O_4_) and O–Metal (from COFs) verifies Fe_3_O_4_ incorporation in the composites.

As shown in Figs. 1g‒i and S11, the Ni 2*p*, Co 2*p*, and Fe 2*p* peaks in s/m-Fe_3_O_4_@Ni‑Salen COF, s/m-Fe_3_O_4_@Co‑Salen COF, and s/m-Fe_3_O_4_@Fe‑Salen COF shift to higher binding energies by 0.16/0.23, 0.26/0.35, and 0.25/0.31 eV, respectively. The positive binding energy shift indicates a reduced local electron density around Ni, Co, and Fe centers, which originates from strong electronic interactions with Fe_3_O_4_ within the MMFR-C. Notably, m-Fe_3_O_4_ induces a larger positive shift than s-Fe_3_O_4_. This trend arises from the stronger intrinsic magnetic field of multi-domain Fe_3_O_4_, which amplifies interfacial electronic interactions in the bioinspired MMFR-C and enhances electron withdrawal from the metal sites. Notably, the shift magnitude exhibits a strong metal-dependent behavior. The Zn 2*p* signal in s/m-Fe_3_O_4_@Zn‑Salen COF shows negligible binding energy variation, owing to the stable *d*^10^ configuration, non-magnetic nature, and weak electron-donating character of Zn^2+^, which lead to minimal electronic coupling with Fe_3_O_4_ [33]. In sharp contrast, distinct positive shifts are observed for magnetic metal centers (Ni, Co, Fe). The shift increases in the order Ni < Co≈Fe, consistent with the increasing number of unpaired *d* electrons and thus the stronger intrinsic magnetic moment of the metal center. These results demonstrate that the magnetic moment of the active metal site and the magnetic domain size of Fe_3_O_4_ act in synergy to modulate interfacial electron transfer within MMFR-C. The gradient electronic regulation induced by different Fe_3_O_4_ domains directly tailors the electron density of active metal centers, which further dictates the selective switching between 2e^−^ and 4e^−^ ORR pathways.

### Magnetic Characterization

**Fig. 2:**
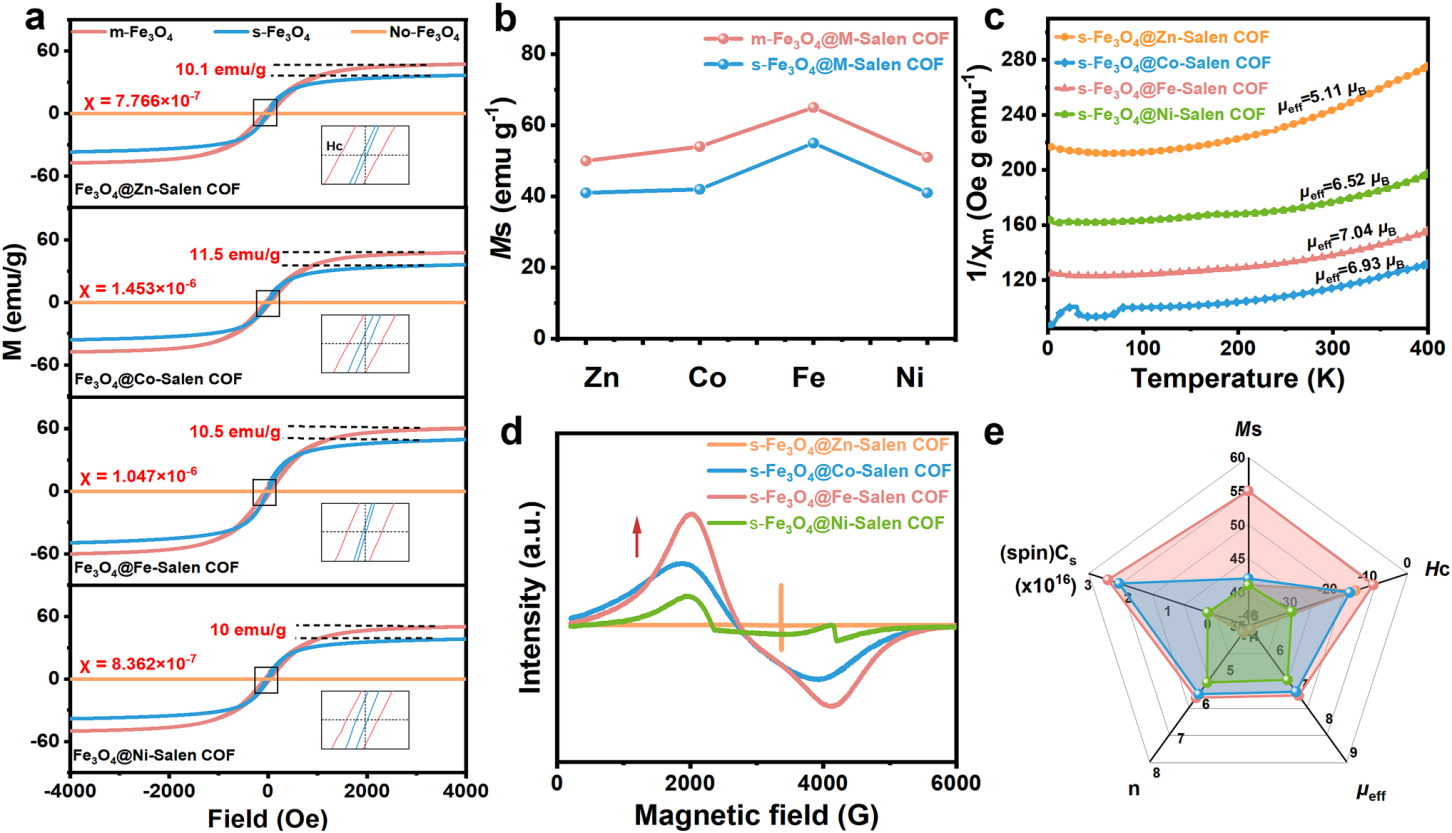
Magnetic characterization of the composites. **a** Magnetic hysteresis loops and **b** the saturation magnetization (*M*s) of catalysts at room temperature of MSA-Salen COFs and s/m-Fe_3_O_4_@MSA-Salen COFs. **c** ZFC curves related to the magnetic susceptibility of s-Fe_3_O_4_@MSA-Salen COFs. **d** EPR spectra of s-Fe_3_O_4_@MSA-Salen COFs. **e** Radar chart depicting magnetic characteristics of different metal materials

To further elucidate the electronic structures of MSA-Salen COFs and MMFR-C, magnetic hysteresis loops were measured at room temperature. MSA-Salen COFs show linear, reversible, and monotonic magnetic responses, consistent with their intrinsic paramagnetic character (Fig. S12a) [34]. The calculated magnetic susceptibilities (*χ*) for Zn‑, Ni‑, Co‑, and Fe‑Salen COF are 7.766 × 10^−7^, 8.362 × 10^−7^, 1.453 × 10^−6^, and 1.047 × 10^−6^, respectively. Notably, clear magnetic domain behaviors are distinguished in MMFR-C (Fig. 2a), the 10 nm Fe_3_O_4_-based composite shows negligible hysteresis, characteristic of single-domain behavior, whereas the 150 nm sample exhibits obvious hysteresis, typical of multi-domain behavior [35, 36 ]. Accordingly, m-Fe_3_O_4_@MSA-Salen COFs display significantly higher saturation magnetization (*Ms*) than s-Fe_3_O_4_@MSA-Salen COFs, confirming a strong correlation between magnetic domain structure and saturation magnetization (Figs. 2b and S12b, c). These results demonstrate that the Fe_3_O_4_ cores not only endow the MMFR-C with tunable magnetic properties but also serve as built-in molecular magnetic field regulators to modulate the electronic structure of metal centers.

Zero-field cooling (ZFC) variable-temperature magnetization measurements further suggest that MSA-Salen COFs are paramagnetic (Fig.  S13). As shown in Figs. 2c and S13a, b, the magnetic susceptibilities (*χ*_m_) of MMFR-C are significantly higher than those of their MSA-Salen COF counterparts. According to the Curie–Weiss law [*χ*_m_ = *C*/(*T−θ*), where *C* is the Curie constant, and *θ* is the Curie temperature] [37], the effective magnetic moment (*μ*_eff_) can be derived from the relation *μ*_eff_ = (8*C*)^1/2^ and is further correlated with the number of unpaired electrons (*n*) via *μ*_eff_ = [*n*(*n* + 2)]^1/2^. The calculated *μ*_eff_ for s‑Fe_3_O_4_@Zn-Salen COF, s‑Fe_3_O_4_@Co-Salen COF, s‑Fe_3_O_4_@Fe-Salen COF, and s‑Fe_3_O_4_@Ni-Salen COF are 5.11, 6.93, 7.04, and 6.52 *μ*_*B*_, respectively, all significantly higher than those of the corresponding pristine Zn-Salen COF (0.097 *μ*_*B*_), Co-Salen COF (0.37 *μ*_*B*_), Fe-Salen COF 0.216 *μ*_*B*_), and Ni-Salen COF (0.123 *μ*_*B*_). A higher *μ*_eff_ reflects a larger effective magnetic moment and increased unpaired electron [38]. The enhanced *μ*_eff_ in s‑Fe_3_O_4_@MSA-Salen COFs further validates that MMFR-C can efficiently modulate the spin state of active metal centers, with the magnetic Fe_3_O_4_ core serving as a built-in field source that exerts a more significant effect on metals with stronger intrinsic magnetism. These results demonstrate that the bioinspired MMFR-C enables controllable spin polarization of single-atom sites via internal magnetic field regulation, laying the structural foundation for switchable ORR pathways.

Electron spin resonance (ESR) spectra reveal a distinct resonance signal for MSA-Salen COFs (Fig. S14), indicative of unpaired electrons inherent to these frameworks. Relative to pristine MSA-Salen COFs, the s‑Fe_3_O_4_@MSA-Salen COF composites in the bioinspired MMFR-C series exhibit a markedly enhanced ESR response (Fig. 2d). This intensified signal signifies an increased population of unpaired electrons, confirming that the Fe_3_O_4_-derived built-in magnetic field within MMFR-C effectively modulates the electronic structure of atomic metal sites [39]. Quantitative ESR analysis yields spin concentrations of 0, 1.81 × 10^14^, 2.24 × 10^16^, and 2.52 × 10^16^ spin g^−1^ for s‑Fe_3_O_4_@Zn‑, Ni‑, Co‑, and Fe‑Salen COF, respectively (Table S3). In agreement with magnetic susceptibility and XPS observations, this modulation displays a pronounced metal-dependent trend, with a more substantial effect observed for metals possessing larger intrinsic magnetic moments (Fe, Co) (Fig. 2e). These characterized results demonstrate that the MMFR-C platform enables magnetic field-mediated spin polarization of active metal centers, establishing a direct correlation between microscopic electronic/spin states and macroscopic magnetic properties.

### Electrochemical Performance of ORR

**Fig. 3:**
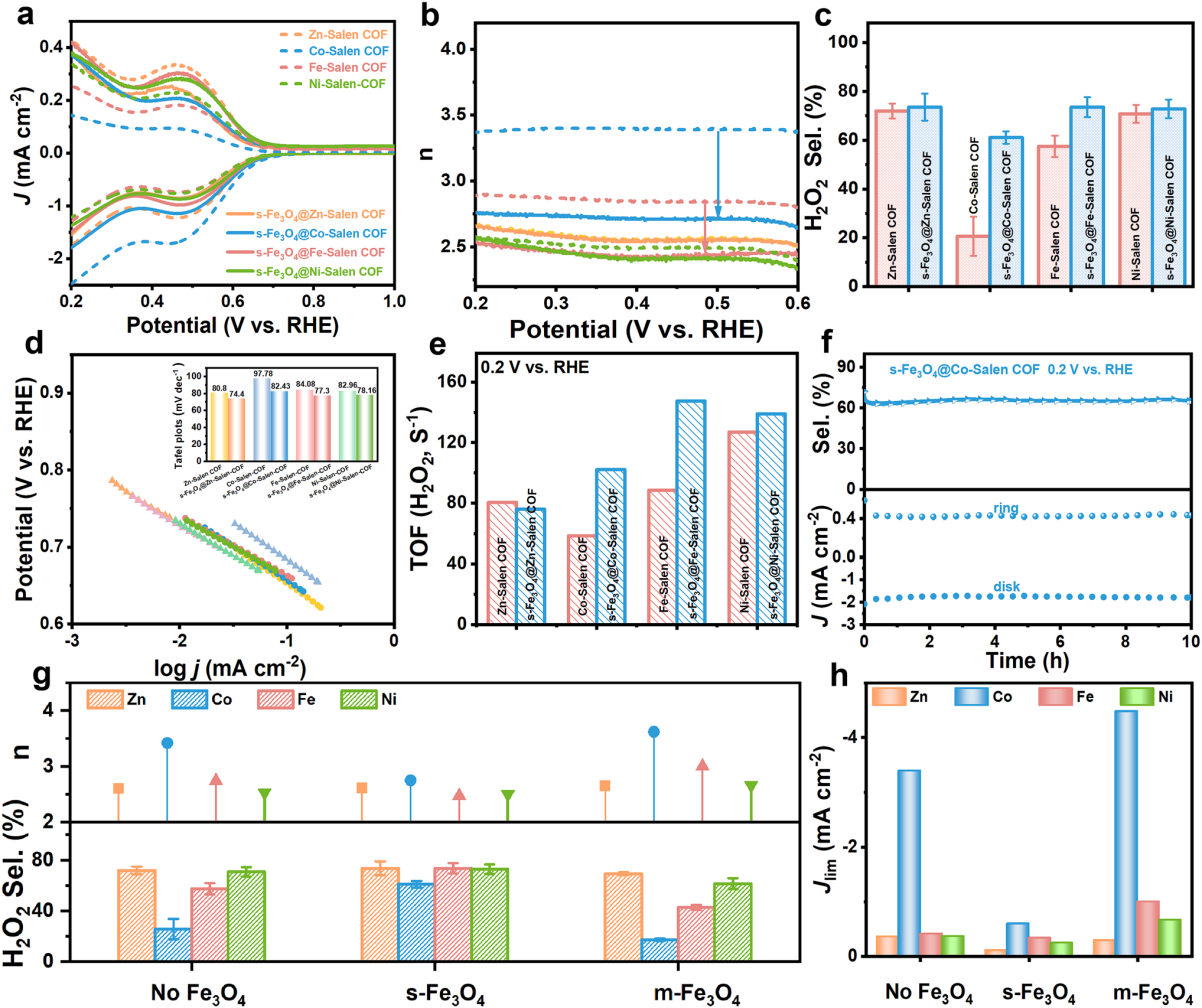
**a** LSV curves of the synthesized catalysts at 1600 rpm, recorded at a scan rate of 10 mV s^−1^ in 0.1 M KOH. **b** Electron transfer number (n) and **c** H_2_O_2_ selectivity of the catalysts, derived from the LSV data. **d** Tafel plots and **e** TOF values at 0.2 V vs. RHE of MSA-Salen COF and s-Fe_3_O_4_@MSA-Salen COF. **f** Chronoamperometry measurement of s-Fe_3_O_4_@Co-Salen-COF for 36,000 s at 0.2 V vs. RHE. **g** Comparison chart of the n and H_2_O_2_ selectivity, and **h** limiting current diagram of H_2_O_2_RR for MSA-Salen COFs and s/m- Fe_3_O_4_@MSA-Salen COFs

The ORR electrocatalytic activities of MSA-Salen COFs and the corresponding MMFR-C were evaluated using a standard three‑electrode configuration in O_2_‑saturated 0.1 M KOH at room temperature. CV curve shows well-defined reduction peaks for MSA-Salen COFs and s‑Fe_3_O_4_@MSA-Salen COFs in O_2_‑saturated electrolyte, with no obvious response in N_2_‑saturated solution, verifying their intrinsic ORR electrocatalytic activity (Fig. S15). As depicted in Fig. 3a, linear sweep voltammetry (LSV) demonstrates that s-Fe_3_O_4_@MSA-Salen COFs exhibit significantly enhanced ring current relative to pristine MSA-Salen COFs, underscoring the key role of magnetic Fe_3_O_4_ incorporation in regulating the ORR pathway and catalytic selectivity within the bioinspired MMFR-C platform.

RRDE measurements were employed to quantify H_2_O_2_ selectivity and electron transfer number, with a collection efficiency of 0.37 (Fig. S16). The electron transfer numbers for Zn‑, Ni‑, Co‑, and Fe‑Salen COF were determined to be 2.61, 2.53, 3.42, and 2.74, respectively, consistent with Koutecky–Levich (K–L) analysis (Figs. S17 and S18). The corresponding single-domain Fe_3_O_4_‑integrated MMFR-C (s‑Fe_3_O_4_@MSA-Salen COFs) exhibited n values of 2.60, 2.50, 2.75, and 2.47, accompanied by high H_2_O_2_ selectivity of 73.5, 72.8, 63.9, and 77.9%, respectively (Fig. 3a–c). These results unambiguously highlight the indispensable role of single-domain Fe_3_O_4_ in governing ORR pathway selectivity within the bioinspired MMFR-C by favoring the selective 2e^−^ ORR route. Notably, within the Co‑Salen COF-derived MFR-C, the incorporation of single-domain Fe_3_O_4_ triggers a distinct catalytic pathway reversal from the intrinsic 4e^−^ process to the desired 2e^−^ route, achieving an over twofold enhancement in H_2_O_2_ selectivity.

Notably, the regulatory effect imposed by single‑domain Fe_3_O_4_ is far more prominent in Co‑ and Fe‑Salen COF-based systems than in Zn‑ and Ni‑based counterparts. Such metal‑dependent divergence originates from the stronger intrinsic magnetism and higher unpaired electron density in Co‑ and Fe‑centered MMFR-C (Fig. 2e), which confer superior sensitivity to the built‑in magnetic field derived from single‑domain Fe_3_O_4_. This specific magnetic coupling within the bioinspired MMFR-C tailors the spin polarization of active metal sites, thereby fine‑tuning the adsorption strength toward critical ORR intermediates and governing the overall pathway selectivity.

A lower Tafel slope is favorable for improving both the selectivity and efficiency of the 2e^−^ ORR pathway [40]. The Tafel slopes of s-Fe_3_O_4_@Zn-Salen COF, s-Fe_3_O_4_@Co-Salen COF, s-Fe_3_O_4_@Fe-Salen COF, and s-Fe_3_O_4_@Ni-Salen COF are 74.4, 82.43, 77.3, and 78.16 mV dec^−1^, respectively, which are uniformly reduced compared with the pristine Zn-Salen COF (80.8 mV dec^−1^), Co-Salen COF (97.78 mV dec^−1^), Fe-Salen COF (84.08 mV dec^−1^), and Ni-Salen COF (82.96 mV dec^−1^) (Fig. 3d). This consistent reduction directly demonstrates that single-domain Fe_3_O_4_ incorporation enhances the ORR electrocatalytic activity and accelerates reaction kinetics within the MMFR-C platform. Furthermore, the double-layer capacitance (*C*_dl_) was evaluated through CV measurements within the non-Faradaic region at various scan rates (Fig. S19), which is directly proportional to electrochemically active surface area (ECSA). As shown in Fig. S20, MMFR-C composites (s‑Fe_3_O_4_@MSA‑Salen COFs) exhibit larger *C*_dl_ values than pristine MSA-Salen COFs, confirming that single-domain Fe_3_O_4_ in MMFR-C facilitates the exposure of more accessible active sites. Electrochemical impedance spectroscopy (EIS) measurements (Figs. S21 and S22) reveal that MMFR-C composites display smaller high-frequency semicircle radii than pristine COFs. This indicates that the synergistic interaction between single-domain Fe_3_O_4_ and metal single-atom COFs within MMFR-C effectively promotes charge transfer capability, further optimizing ORR performance.

To evaluate intrinsic catalytic activity, the H_2_O_2_-selective mass activity (MA(H_2_O_2_), Fig. S23) and turnover frequency (TOF) at 0.2 V vs. RHE (Fig. 3e) were analyzed. The single-domain Fe_3_O_4_-integrated MMFR-C (s‑Fe_3_O_4_@MSA-Salen COFs) exhibited substantially superior MA(H_2_O_2_) and TOF compared to their pristine MSA-Salen COF counterparts, confirming that single-domain Fe_3_O_4_ within MMFR-C enhances intrinsic 2e^−^ ORR activity. Notably, the Co‑Salen COF-derived MMFR-C (s‑Fe_3_O_4_@Co‑Salen COF) achieved a remarkably high TOF of 102.14 s^−1^ and an MA(H_2_O_2_) of 93.61 mA mg^−1^ at 0.2 V vs. RHE, demonstrating exceptional 2e^−^ ORR catalytic performance. Stability is a critical criterion for practical ORR applications [41]. Thus, the long-term stability of s‑Fe_3_O_4_@Co‑Salen COF was evaluated via chronoamperometry in O_2_-saturated 0.1 M KOH at 0.2 V vs. RHE (Fig. 3f). Negligible decay in both disk and ring currents after 10 h of continuous operation confirmed the excellent long-term catalytic stability of this MMFR-C. We harvested s-Fe_3_O_4_@Co-Salen COF catalysts from the electrode surface and further carried out XRD and XPS characterizations. As revealed by XRD results (Fig. S24), the diffraction peak positions of the catalyst after stability cycling are highly consistent with those of the fresh sample, and no characteristic peaks corresponding to new phases are detected. This demonstrates that the intrinsic crystal structure of Fe_3_O_4_ and the skeleton structure of COF are well retained after long-term testing. Correspondingly, XPS analysis (Fig. S24b, c) further confirms that no obvious shifts occur in the binding energies and peak profiles of Co 2*p*, O 1* s*, and N 1* s* spectra after the stability test. In addition, cobalt species still predominantly maintain the + 2 oxidation state, which strongly evidences that the chemical valence state of active sites possesses excellent stability during prolonged electrocatalytic operation.

To investigate the effect of magnetic domains on ORR selectivity, the electrocatalytic performance of multi-domain Fe_3_O_4_-integrated MMFR-C (m‑Fe_3_O_4_@MSA-Salen COFs) was evaluated. As shown in Fig. S25, the half-wave potentials of m‑Fe_3_O_4_@Co‑, Fe‑, and Ni‑Salen COFs all undergo a positive shift relative to their pristine MSA-Salen COF counterparts. RRDE measurements (Figs. 3g and S26) yield electron transfer numbers of 3.67, 3.02, and 2.66 for m‑Fe_3_O_4_@Co‑, Fe‑, and Ni‑Salen COFs, respectively, indicating a preference for the 4e^−^ ORR pathway. These results confirm that multi-domain Fe_3_O_4_ acts as a key regulatory component within MMFR-C, facilitating the preferential occurrence of the 4e^−^ ORR pathway.

To further clarify the role of s/m-Fe_3_O_4_ in MMFR-C, the intrinsic ORR performance of bare s/m-Fe_3_O_4_ particles was evaluated (Fig. S27). Bare Fe_3_O_4_ favors a near-3e^−^ pathway, confirming that the tunable ORR selectivity of MMFR-C stems from the synergistic interaction between Fe_3_O_4_ and MSA-Salen COFs rather than the standalone catalytic activity of Fe_3_O_4_. Notably, RRDE measurements of s/m-Fe_3_O_4_ exhibit significant disk-ring current asymmetry, likely due to competitive side reactions causing a continuous increase in disk current. To gain additional insights into MMFR-C’s ORR selectivity toward H_2_O_2_ production, polarization curves for the hydrogen peroxide reduction reaction (H_2_O_2_RR) were recorded in Ar-saturated 0.1 M KOH containing 50 mM H_2_O_2_ (Fig. S28), where current density comparison quantifies the capability to inhibit H_2_O_2_ decomposition. As depicted in Fig. 3h, single-domain Fe_3_O_4_-integrated MMFR-C (s‑Fe_3_O_4_@MSA-Salen COFs) exhibits the lowest limiting current densities, endowing them with excellent ability to suppress H_2_O_2_ decomposition during the 2e^−^ ORR process and thus favoring high-concentration H_2_O_2_ generation [42], which is consistent with the preceding RRDE results.

To optimize the loading amount, we systematically investigated Fe_3_O_4_ contents ranging from 10 to 30 wt%. XRD results showed that as the Fe_3_O_4_ content increased, the diffraction peak intensity of the COF gradually decreased (Fig. S29a, b). When the loading of multi-domain magnetite was further increased to 35 wt%, the crystallinity of the COF was significantly degraded; therefore, only the 10 − 30 wt% range was selected for electrochemical testing (Fig. S30). The test results indicated that the electron transfer number of the multi-domain material remained stable at approximately 3.67 within the 10 − 30 wt% range, while the single-domain material achieved the optimal regulation effect at 20 wt% (electron transfer number of 2.72). Considering these factors, 20 wt% was uniformly chosen as the representative loading. This content achieves saturated pathway regulation in each system while avoiding COF crystal quality degradation caused by excessively high loading.

The yield of H_2_O_2_ was quantitatively determined using the K_2_TiO(C_2_O_4_)_2_ colorimetric method (Figs. S31–S35) [43]. H₂O₂ yields at different constant currents showed that s-Fe_3_O_4_@Zn-Salen COF, s-Fe_3_O_4_@Co-Salen COF, s-Fe_3_O_4_@Fe-Salen COF, and s-Fe_3_O_4_@Ni-Salen COF achieved H_2_O_2_ production rates of 77.93, 93.35, 119.74, and 76.64 mmol g^−1^ h^−1^ at 0.2 V vs. RHE, with corresponding Faradaic efficiencies (FE) of 74.02, 61.27, 73.58, and 67.78%, respectively. It is indicated that the MMFR-C exhibits significantly enhanced H_2_O_2_ production rates and higher Faradaic efficiencies compared to their pristine MSA-Salen COF. This improvement is particularly pronounced for the Co- and Fe-based systems, indicating a marked enhancement in both selectivity and activity toward the two-electron oxygen reduction pathway for H_2_O_2_ generation. These findings are consistent with and corroborate the earlier LSV results. Notably, the s-Fe_3_O_4_@Fe-Salen COF composite achieves an H_2_O_2_ production rate as high as 132 mmol g^−1^ h^−1^ at 0.3 V vs. RHE. This enhancement is attributed to the moderate spin polarization effect induced by the magnetic s-Fe_3_O_4_ nanoparticles on the Fe-Salen COF, which promotes electron transfer and optimizes the adsorption of key reaction intermediates.

Meanwhile, to validate the above trends, the H_2_O_2_ production rate and FE of multi-domain Fe_3_O_4_‑integrated MMFR-C (m‑Fe_3_O_4_@Co/Fe/Ni‑Salen COFs) were quantified at 0.3 V vs. RHE. As displayed in Fig. S36a–c, both H_2_O_2_ yield and FE decrease monotonically with increasing Fe_3_O_4_ domain size. This result further confirms that large multi-domain Fe_3_O_4_ within MMFR-C favors the competing 4e^−^ ORR pathway at the expense of the 2e^−^ route for H_2_O_2_ generation, which is fully consistent with the earlier LSV measurements.

Given the outstanding 2e^−^ ORR performance and high H_2_O_2_ productivity of s‑Fe_3_O_4_@Fe‑Salen COF and s‑Fe_3_O_4_@Co‑Salen COF within the MMFR-C series, their potential for practical environmental remediation was further explored [44]. As H_2_O_2_ serves as a key oxidant for advanced oxidation processes, the in situ electrogenerated H_2_O_2_ was employed to degrade Rhodamine B as a model organic pollutant. H_2_O_2_ generated at 0.2 V vs. RHE over 3 h in an H‑cell was collected for degradation tests. UV–Vis spectra and visual inspection confirmed complete degradation of the dye within 1 h (Fig. S37a, b), demonstrating efficient in situ H_2_O_2_ production by these MMFR-C.

### Insight into the Catalytic Mechanism

**Fig. 4:**
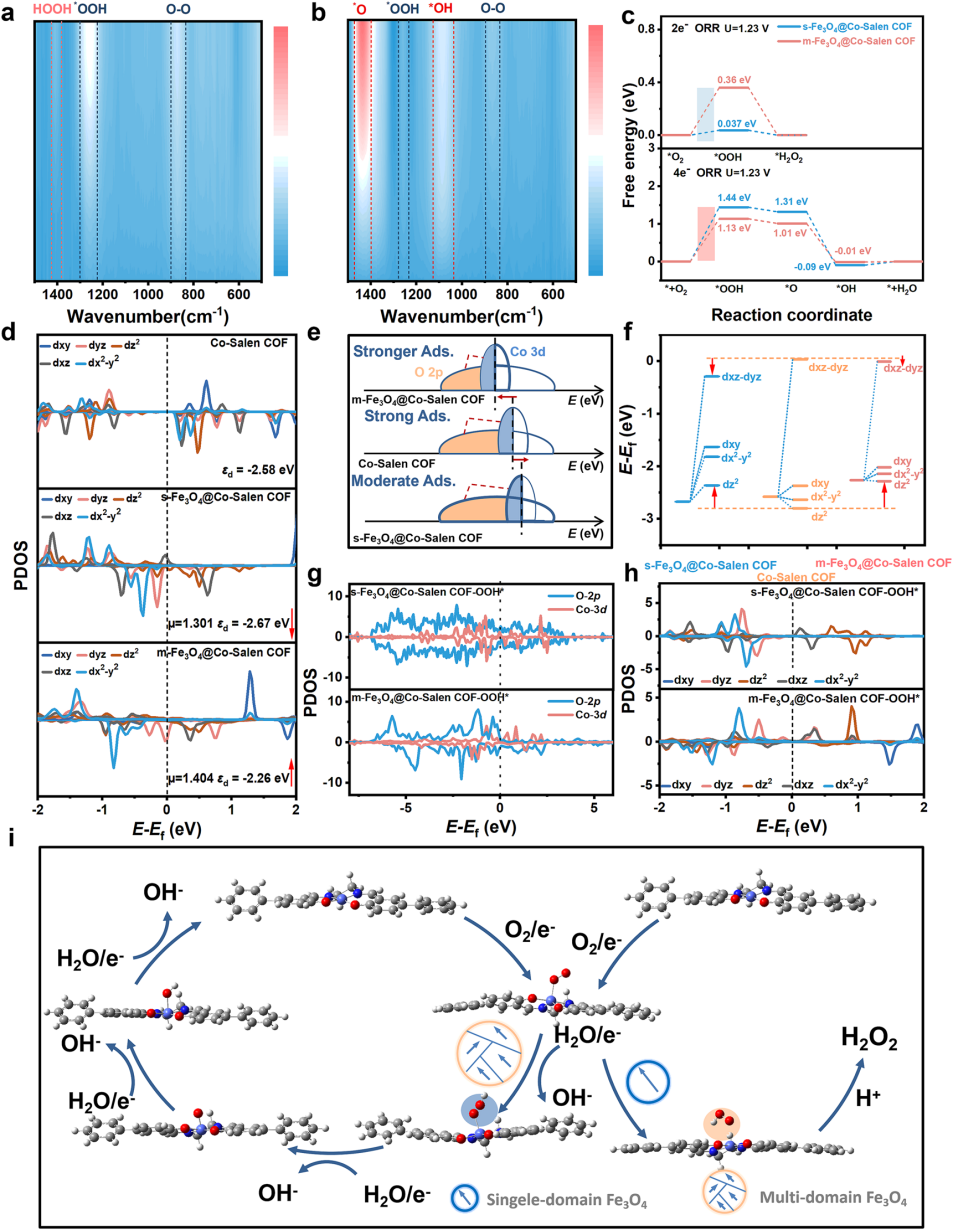
In situ ATR-FTIR spectra of **a** s-Fe_3_O_4_@Co-Salen-COF and **b** m-Fe_3_O_4_@Co-Salen-COF catalysts in O_2_-saturated 0.1 M KOH solution (pH 13) at different potentials from 0.8 to 0 V vs. RHE. **c** Free energy profiles for 2e^–^ ORR and 4e^–^ ORR of s-Fe_3_O_4_@Co-Salen-COF and m-Fe_3_O_4_@Co-Salen-COF. **d** Projected density of states (PDOS) of Co-Salen-COF, s-Fe_3_O_4_@Co-Salen-COF and m-Fe_3_O_4_@Co-Salen-COF. **e** Energy band structure diagrams. **f** Band energy structures of Co-Salen-COF, s-Fe_3_O_4_@Co-Salen-COF, and m-Fe_3_O_4_@Co-Salen-COF. **g** PDOS of the Co center and the O atom in OOH* intermediate for s-Fe_3_O_4_@Co-Salen-COF and m-Fe_3_O_4_@Co-Salen-COF. **h** Computed PDOS of s-Fe_3_O_4_@Co-Salen-COF and m-Fe_3_O_4_@Co-Salen-COF after OOH* adsorption. **i** Schematic ORR process on the N_2_–M–O_2_ site of MSA-Salen-COF

 In situ attenuated total reflectance Fourier-transform infrared spectroscopy (ATR-FTIR) spectra reveal that for single-domain MMFR-C (s-Fe_3_O_4_@Co-Salen COF), characteristic peaks at ~ 1250 and 872 cm^−1^, consistent with *OOH stretching vibrations [45], are significantly intensified as the potential decreases (Figs. 4a and S38a). A weak H_2_O_2_ signal at 1407 cm^−1^ further confirms the dominance of the 2e^−^ pathway. By contrast, multi-domain MMFR-C (m-Fe_3_O_4_@Co-Salen COF) shows markedly diminished *OOH signals, with new peaks at 1440 and 1086 cm^−1^ assignable to *O and *OH, typical of the 4e^−^ pathway (Figs. 4b and S38b) [46]. Spin-polarized density functional theory (DFT) calculations were performed to elucidate the spin-dependent ORR kinetics over single-domain and multi-domain MMFR-C (Fig. S39). Gibbs free energy profiles identify *OOH formation as the rate-determining step for the 2e^−^ pathway (Fig. 4c) [47]. Single-domain MMFR-C (s-Fe_3_O_4_@Co-Salen COF) delivers an ultralow-energy barrier of only 0.037 eV for *OOH generation, with *OOH stabilized at a higher free energy state to favor desorption as H_2_O_2_ instead of O–O cleavage. Multi-domain MMFR-C (m-Fe_3_O_4_@Co-Salen COF) presents a substantially higher barrier of 0.36 eV for the same step, while stabilizing *OOH at a lower free energy level to facilitate its dissociation into *O/*OH. These thermodynamic distinctions establish single-domain MMFR-C as a selective 2e^−^ ORR catalyst and multi-domain MMFR-C as a preferential 4e^−^ ORR catalyst with promoted O–O bond cleavage.

The projected density of states (PDOS) analysis of the Co 3*d* orbitals (Fig. S40) reveals that the m-Fe_3_O_4_@Co-Salen COF exhibits more pronounced asymmetry in the electron distribution across different *d*-orbitals, highlighting stronger spin polarization features compared to the s-Fe_3_O_4_@Co-Salen COF, which shows relatively weaker spin polarization. This discrepancy arises from the distinct strengths of internal magnetic fields within the two MMFR-C variants, which induce varying degrees of polarization at the Co active sites.

Integrated experimental and theoretical results identify *OOH adsorption strength as a key descriptor for ORR selectivity, which is closely linked to the *d*-band center of the Co active site [48]. As shown in Fig. 4d, the calculated *d*-band center value for Co-Salen COF is –2.58. Single-domain MMFR-C (s-Fe_3_O_4_@Co-Salen COF) slightly downshifts the *d*-band center to − 2.67 eV, weakening the CO–OOH interaction to an optimal strength. This favors *OOH formation and protonation while suppressing O–O cleavage, directing the reaction to the 2e^−^ pathway for H_2_O_2_ production. Multi-domain MMFR-C upshifts the *d*-band center to − 2.26 eV via stronger spin polarization, strengthening *OOH adsorption, and promoting O–O bond cleavage to drive the 4e^−^ route. These trends are fully supported by free energy barriers, in situ ATR-FTIR, and electrochemical performance. These results demonstrate that the magnetic domain in MMFR-C enables precise tuning of the spin polarization and *d*-band center, allowing selective and controllable switching between 2e^−^ and 4e^−^ ORR pathways by optimizing *OOH adsorption energy (Fig. 4e).

Magnetic catalysts can effectively overcome spin-forbidden O_2_ activation via spin-polarized charge transfer, lowering the kinetic barrier for the initial reaction step [49]. Given side-on O_2_ adsorption at the Co single-atom site, M − O bond formation relies on hybridization between O_2_ π* orbitals and *dz*^2^ and *dxz*-*dyz* orbitals, whose extent directly governs *O_2_ activation (Fig. 4f and Table S4). Single-domain MMFR-C (s-Fe_3_O_4_@Co-Salen COF) induces only a minor upshift of the Co *dz*^2^ orbital (0.007 eV) and a downshift of the *dxz*-*dyz* orbitals (0.326 eV). Moderate spin polarization enables initial O_2_ activation while suppressing electron back-donation to O_2_ π* orbitals, stabilizing *OOH and inhibiting O–O cleavage to favor the 2e^−^ pathway [24]. Multi-domain MMFR-C (m-Fe_3_O_4_@Co-Salen COF) delivers a pronounced upshift of the Co *dz*^2^ orbital (0.228 eV), strengthening orbital hybridization and improving spin matching between Co-3*d* and O-2*p*. This facilitates efficient overcoming of the spin prohibition during O_2_ activation, paving the way for subsequent O–O bond cleavage and thus the 4e^‒^ pathway. DFT calculations reveal the charge transfer between *OOH and the Co active site. DFT analysis further shows that single-domain MMFR-C exhibits a smaller Bader charge (1.25 e^−^) than multi-domain MMFR-C (1.26 e^−^), weakening Co–*OOH binding and promoting *OOH desorption for selective H_2_O_2_ generation.

PDOS calculations were performed for s-Fe_3_O_4_@Co-Salen COF and m-Fe_3_O_4_@Co-Salen COF following *OOH adsorption. Hybridization between the Co *d*-band and O *p*-band emerges upon oxygen adsorption, and the energy gap between their band centers reflects the metal-intermediate interaction strength. The gap is determined to be 4.60 eV for single-domain MMFR-C and 4.64 eV for multi-domain MMFR-C (Fig. 4g) [50]. The narrower gap in single-domain MMFR-C indicates a moderate Co–*OOH binding strength, which is optimal for H_2_O_2_ generation.

As shown in Fig. 4h, single-domain MMFR-C forms the Co− *OOH bond primarily via the *dyz* orbital, which is energetically downshifted relative to the Fermi level (*E*_f_) [51]. This, along with a lower *d*-band center of –2.03 eV (Fig. S41), indicates moderate *OOH adsorption, facilitating *OOH hydrogenation and H_2_O_2_ desorption to favor the 2e^−^ pathway. Multi-domain MMFR-C is dominated by the *dyz* orbital near *E*_f_, with a higher *d*-band center of − 1.64 eV that strengthens *OOH adsorption. This stabilizes *OOH and reduces the O–O bond cleavage barrier, driving the formation of *O/*OH intermediates and directing the ORR to the 4e^−^ route.

Figure 4i illustrates the complete catalytic cycles for the 2e^−^ and 4e^−^ ORR pathways over the MMFR-C. The reaction commences with spontaneous adsorption of ground-state O_2_ at the coordinatively unsaturated metal active center, followed by proton-coupled electron transfer to generate the *OOH intermediate, which is the key branching point determining ORR selectivity. In single-domain MMFR-C, moderate, uniform spin polarization from the internal magnetic field modulates the metal site’s electronic structure and orbital hybridization, optimizing *OOH adsorption energy to a thermodynamically favorable range. This moderate binding stabilizes the metal–OOH adduct, enabling preferential protonation of the proximal oxygen coordinated to the metal center. This promotes H_2_O_2_ reductive elimination and metal site regeneration, selectively channeling the reaction toward the 2e^−^ pathway for high-yield H_2_O_2_ production. In multi-domain MMFR-C, stronger, localized magnetic ordering induces intensive spin polarization at the metal site, significantly strengthening the metal–*OOH interaction. Enhanced orbital overlap and charge transfer favor protonation of the distal oxygen in metal–OOH, triggering O–O bond cleavage to form a metal-bound *O intermediate and free H_2_O. Subsequent proton-coupled electron transfer steps drive the reaction toward the complete 4e^−^ pathway for H_2_O formation.

## Conclusions

In summary, this work establishes a novel strategy by constructing bioinspired MMFR-C to realize on-demand, reversible switching between the 2e^−^ and 4e^−^ ORR pathways. A mild, uniform endogenous magnetic field from single-domain MMFR-C optimizes orbital hybridization and *OOH adsorption energy, suppressing O–O bond cleavage to favor H_2_O_2_ generation. Representative single-domain MMFR-C (s-Fe_3_O_4_@Co-Salen COF) achieves 63.9% H_2_O_2_ selectivity with an electron transfer number of 2.72, far exceeding pristine Co-Salen COF (26% selectivity, *n* = 3.48). By contrast, a stronger magnetic field in multi-domain MMFR-C (m-Fe_3_O_4_@Co-Salen COF) optimizes the *d*-band center, lowers spin transition barriers, and enhances intermediate adsorption, promoting O–O bond cleavage and directing the ORR to the 4e^−^ pathway (*n* = 3.67). This work demonstrates that MMFR-C enables precise on-demand ORR pathway control, providing a new paradigm for designing selectivity-tunable ORR electrocatalysts.

## Supplementary Information

Below is the link to the electronic supplementary material.

Supplementary file1 (DOCX 28102 KB)
