# Supplementary material for: Bioinspired Molecular Magnetic Field-Responsive Catalyst for On-Demand Switching of 2e− and 4e− Oxygen Reduction
Source: Nanomicro Lett. 2026 Aug 10;19:26. doi: 10.1007/s40820-026-02319-0 (PMC13454089; doi:10.1007/s40820-026-02319-0)
Supplement: Supplementary file 1 [file 40820_2026_2319_MOESM1_ESM.docx]

Supporting Information for

**Bioinspired Molecular Magnetic Field Responsive Catalyst for On-Demand Switching of 2e⁻ and 4e⁻ Oxygen Reduc****tion**

Boying Zhang^‡, 1^, Qiaoling Guo^‡, 1^, Haochuan Li^1^, Yue Wang^1^, Qing Li^1^, Haining Liu^*1, 2^, Shanlin Qiao*^, 1, 2, 3^

^1^ College of Chemistry and Pharmaceutical Engineering, Hebei University of Science and Technology, Shijiazhuang 050018, P. R. China

^2^ Hebei Research Center of Pharmaceutical and Chemical Engineering, Shijiazhuang 050018, P. R. China

^3^ Hebei Engineering Research Center of Organic Solid Photoelectric Materials for electronic information, Shijiazhuang 050018, P. R. China

‡ Boyingzhang and Qiaoling Guo contributed equally to this work.

*Corresponding authors. E-mails: [liuhn@hebust.edu.cn](mailto:liuhn@hebust.edu.cn) (Haining Liu); [ccpeslqiao@hebust.edu.cn](mailto:ccpeslqiao@hebust.edu.cn) (Shanlin Qiao)

**S1 Supplementary Text**

***S1.1 Materials***

1,3,5-tris(4-hydroxy-5-formylphenyl)benzene, N, N-dimethylformamide, ethanediamine, ethanol, and acetone were purchased from Damao Chemical Reagent Factory (Tianjin, China). Fe_3_O_4_, Potassium titanium oxalate K_2_TiO(C_2_O_4_)_2_, Zn(OAC)_2_·2H_2_O, Cu(OAC)_2_·H_2_O, Ni(OAC)_2_·4H_2_O, Co(OAC)_2_·2H_2_O, Fe(OAC)_2_·H_2_O, and Mn(OAC)_2_·4H_2_O were obtained from Aladdin Industrial Corporation (Shanghai, China). All chemicals were used without further purification.

***S1.2 Electrochemical measurements***

The electrochemical tests were conducted on an RRDE on a CHI 760E workstation, coupled to an RRDE rotator: a glassy carbon disc electrode (Ø = 5.61 mm), a Pt ring electrode (Ø inner = 6.25 mm, Ø outer = 7.9 mm), and a graphite rod was used as counter electrode, and Ag/AgCl as reference electrode. All potentials were converted to the reversible hydrogen electrode (RHE) scale using the Nernst equation E_RHE_ = E_Ag/AgCl_ + 0.197 + 0.0591 × pH. Each catalyst (5 mg) was dispersed into 1 mL solution composed of 9: 10: 1 = ethanol: H_2_O: Nafion (5 wt%) solution, and then the mixed solution was sonicated for 30 mins. 10 μL of ink was loaded onto the RRDE electrode and dried at 500 rpm.

The H_2_O_2_ selectivity, electron-transfer number (n), and Faradaic efficiency (%) were calculated from the following function, respectively:

$$\begin{aligned} \text{H}_{\text{2}}\text{O}_{\text{2}}\text{ selectivity (\%) =200 × }\frac{{\text{I}_{\text{R}}}/\text{N}}{\text{I}_{\text{D}}\text{ + }{\text{I}_{\text{R}}}/\text{N}} \end{aligned}(S1)$$

$$\begin{aligned} \text{n = 4 × }\frac{\text{I}_{\text{D}}}{\text{I}_{\text{D}}\text{-}{\text{I}_{\text{R}}}/\text{N}} \end{aligned}(S2)$$

$$\begin{aligned} \text{Faradaic efficiency(\%)}\text{ = }\frac{\text{100 × }\text{I}_{\text{R}}}{\left| \text{I}_{\text{D}} \right|\text{×N}} \end{aligned}(S3)$$

where *I*_R_ and *I*_D_ are the ring current and disk current. N is the current collection efficiency of the ring electrode.

Cyclic voltammetry (CV) was measured at various scan rates to estimate the electrochemically active surface area (ECSA). The CV curves were recorded in 0.1 M KOH over a potential range where Faradaic reactions are negligible, with scan rates (ν) of 40, 60, 80, 100, 120, 140 mV s^−1^. The capacitance of the double layer (*C*_dl_) was calculated from the slope of the plot of average current density versus scan rate:

$$\begin{aligned} \text{C}_{\text{dl}}\text{ = }\frac{\text{j}}{\text{ν}} \end{aligned}(S4)$$

Where *j* is the average current density, determined from the CV curves.

The EIS measurements were conducted by applying an AC voltage with 5 mV amplitude in a frequency range from 0.01-10^6^ Hz, measured at 0.7 V vs. RHE in 0.1 M KOH.

To aid in verifying the accuracy of the RRDE test, Koutecky-Levich plots were performed. By testing the disk current at 400-1600 rpm, the number of electrons transferred was calculated using the Koutecky-Levich (K-L) equation.

$$\begin{aligned} \frac{\text{1}}{\text{j}}\text{ = }\frac{\text{1}}{\text{j}_{\text{L}}}\text{ + }\frac{\text{1}}{\text{j}_{\text{K}}}\text{= }\frac{\text{1}}{\text{B}\text{ × }\text{ω}^{\text{1/2}}}\text{ + }\frac{\text{1}}{\text{j}_{\text{K}}} \end{aligned}(S5)$$

$$\begin{aligned} \text{B}\text{ = 0.62}\text{nF}\text{C}_{\text{0}}\text{D}_{\text{0}}^{\text{3/2}}\text{ν}^{\text{-1/6}} \end{aligned}(S6)$$

Where *j* is the measured current density, *j*_K_ is the kinetic current density, and *j*_L_ is the diffusion-limiting current density. ω represents the angular velocity of the disk (rad s^-1^), *n* is the number of electrons transferred in the ORR, and F is the Faraday constant (96485 C mol^−1^). *C*_0_ and *D*_0_ denote the bulk concentration of O_2_ (0.84 × 10^−3^ mol cm^−3^) and the diffusion coefficient of O_2_ (1.65 × 10^−5^ cm^2^ s^−1^), respectively. *ν* is the kinematic viscosity of the electrolyte (0.01 cm^2^ s^−1^). All parameters are determined under standard atmospheric pressure at 25 °C.

Mass Activity and Turnover Frequency (TOF) were calculated as follows. The mass activity (mA mg^−1^) values were calculated from the catalyst mass *m* (mg cm^−2^) and the kinetic current density *j_K_* (mA cm^−2^):

$$\begin{aligned} \text{Mass activity}\text{ }\text{(H}_{\text{2}}\text{O}_{\text{2}}\text{) = }\frac{\text{j}_{\text{K}}\text{ × }\text{A}_{\text{geom}}\text{ × }\text{r}_{\text{f}}}{\text{ }\text{m}_{\text{cat}}}\text{ }\text{× }\text{H}_{\text{2}}\text{O}_{\text{2}}\text{ selectivity} \end{aligned}(S7)$$

Where *r*_f_ is the roughness factor, defined as the ratio of the actual surface area of the electrode to its geometric surface area. The TOF values were calculated using the following equation:

$$\begin{aligned} \text{TOF}\text{ = }\frac{\text{j}_{\text{H}\text{2}\text{O}\text{2}}\text{ × }\text{A}_{\text{geom}}}{\text{2}\text{×}\text{F}{\text{×}\text{n}}_{\text{Fe}}} \end{aligned}(S8)$$

Where *A*_geom_ is the surface area of the disk electrode, the number 2 represents 2 electrons per mole of O_2_, F is the Faraday constant (96485 C mol^−1^), *n*_Fe_ is the moles of Fe atoms coated on the electrode calculated from relative element contents in the XPS spectra, and *j*_H2O2_ is the H_2_O_2_ current density calculated using the following equation:

$$\begin{aligned} \text{j}_{\text{H2O2}}\text{ = }\frac{\text{I}_{\text{R}}}{\text{N ×}\text{ A}_{\text{geom}}} \end{aligned}(S9)$$

***S1.3 H_2_O_2_ yield measurement method***

The H_2_O_2_ yield was measured by the classic potassium titanate oxalate color method.4 The basis of this method is the formation of titanium (IV)-peroxide complex in the presence of sulfuric acid. The detailed process of this method could be described as follows: a certain volume (1 mL) of the sample to be measured was added to the as-prepared potassium titanium oxalate K_2_TiO(C_2_O_4_)_2_

solution (0.5 M, 1 mL) and 3 M H_2_SO_4_ solution (1 mL), and then the color of the solution changed to yellow because of the above chromogenic reaction. Next, UV-Vis technology (at 400 nm) was used to determine and calculate the content of the H_2_O_2_ in the samples.

The faradaic efficiency (FE) was calculated using the following equation:

$$\begin{aligned} \text{FE(\%) = }\frac{\text{2 ×C ×V ×F}}{\text{Q}}\text{ ×100\%} \end{aligned}(S10)$$

where *F* is the Faraday constant (96485 C mol^-1^), C is the concentration of generated H_2_O_2_, *V* is the volume of the electrolyte (20 mL), and *Q* is the total charge passed during the electrolysis.

***S1.4 Dye degradation***

A 2 mL aliquot of the electrolyte was acidified with 8 mL of 2 M H_2_SO_4_. Subsequently, 1 mg of FeSO_4_ was added to initiate the Fenton-like reaction for generating -OH radicals. Then, 10 mL of a 10 ppm Rhodamine B (RB) solution was introduced into the mixture. The discoloration of the solution was monitored by recording its color and measuring the UV-vis absorbance at time intervals of 0, 30, 60, and 120 minutes.

***S1.5 In situ ATR-FTIR measurements***

In situ ATR-FTIR measurements were performed on a Nicolet 6700 FTIR 466 spectrometer equipped with an ATR accessory with a diamond window. Typically, A thin layer of graphene film was chemically deposited on the surface of silicon crystals, and then 100 microliters of catalyst ink (10 mg mL^−1^) was dropped onto the graphene film, with the entire device serving as the working electrode. Using platinum as the counter electrode and Ag/AgCl as the reference electrode. The electrolyte was 0.1 M KOH. Before testing, high-purity O_2_ was introduced for 30 minutes until the electrolyte was saturated with O_2_. The infrared spectrum collected at the open-circuit potential was used as the background spectrum.

***S1.6 Computational details***

Spin-polarized density functional theory calculations were performed using the Perdew-Burke-Ernzerhof (PBE) functional and the projector augmented-wave (PAW) potential as implemented in the Vienna Ab Initio Simulation Package (VASP) [S1−S5]. An energy cutoff of 400 eV and a convergence criterion of 10−7 eV were adopted for self-consistent calculations. All structures were relaxed until the total force on each relaxed atom was less than 0.04 eV/Å. The van der Waals interaction was corrected based on the DFT-D3 scheme [S6]. The thickness of the vacuum layer was larger than 15 Å. A Γ-centered 2 × 2 × 1 k-point was adopted. VASPKIT code [S7] and VESTA software [S8] were used for calculation pre-processing and post-processing.

**S2 Supplementary Figures**

Fig. S1 The structure schematic of MSA-Salen COF.

Fig. S2 FT-IR spectrum of THB, Zn-Salen COF, s-Fe_3_O_4_@Zn-Salen COF, and m-Fe_3_O_4_@Zn-Salen COF.

**Fig. S3** P-XRD pattern of a) MSA-Salen COF, b) s-Fe_3_O_4_@Zn-Salen COF, and c) m-Fe_3_O_4_@Zn-Salen COF (Metal=Zn, Fe, Co, Ni).

**Fig. S4** a) N_2_ adsorption/desorption isotherms and b) the pore width distribution plot of Zn-Salen- COF, s-Fe_3_O_4_@Zn-Salen COF, and m-Fe_3_O_4_@Zn-Salen COF.

**Fig. S5** TEM of a) s-Fe_3_O_4_ and b) m-Fe_3_O_4_.

**Fig. S6** HR-TEM of a) s-Fe_3_O_4_@Zn-Salen COF and b) m-Fe_3_O_4_@Zn-Salen COF.

**Fig. S7** HR-TEM of s-Fe_3_O_4_@MSA-Salen COFs.

**Fig. S8** EXAFS fitting curves of Zn-Salen COF.

**Fig. S9** N 1*s* high resolution XPS spectra of MSA-Salen COFs and s/m-Fe_3_O_4_@MSA-Salen COFs.

**Fig. S10** O 1*s* high-resolution XPS spectra of MSA-Salen COFs and s-Fe_3_O_4_@MSA-Salen COFs.

**Fig. S11** High-resolution XPS spectra of Zn 2*p*.

**Fig. S12** Magnetic hysteresis loops of catalysts at room temperature of a) MSA-Salen COFs, b) s-Fe_3_O_4_@MSA-Salen COFs, and c) m-Fe_3_O_4_@MSA-Salen COFs.

**Fig. S13** ZFC curves related to the magnetic susceptibility of MSA-Salen COFs.

**Fig. S14** ZFC curves related to the magnetic susceptibility of MSA-Salen COFs and s-Fe_3_O_4_@MSA-Salen COFs.

**Fig. S15** CV curves of MSA-Salen COF and s-Fe_3_O_4_@MSA-Salen COFs modified glassy carbon electrode in a 0.1 M KOH solution. Scan rate: 10 mV s^−1^.

**Fig. S16** Calibration of the collection efficiency of the bare RRDE in N_2_-saturated 0.05 M Na_2_SO_4_ with 4 mM K_3_[Fe(CN)_6_] solution. (a) LSV curves recorded at different rotation rates. Scan rate: 10 mV s^−1^, *E*_ring_ = 1.3 V vs RHE. (b) The corresponding collection efficiency of RRDE voltammograms as a function of the potential.

**Fig. S17** LSV curves and determined transfer electron number (n) of MSA-Salen COF in O_2_-saturated 0.1 M KOH at different rotation rates.

**Fig. S18** LSV curves and determined transfer electron number (n) of s-Fe_3_O_4_@MSA-Salen COFs in O_2_-saturated 0.1 M KOH at different rotation rates.

**Fig. S19** CV curves of MSA-Salen COF and s-Fe_3_O_4_@MSA-Salen COFs at various scan rates from 20 to 100 mV s^−1^.

**Fig. S20** The summarized *C*_dl_ of MSA-Salen COFs and s-Fe_3_O_4_@MSA-Salen COFs.

**Fig. S21** Nyquist plots of MSA-Salen COFs and s-Fe_3_O_4_@MSA-Salen COFs.

**Fig. S22** Equivalent circuit diagram.

**Fig. S23** Mass activity of H_2_O_2_ production of MSA-Salen COFs and s-Fe_3_O_4_@MSA-Salen COFs.

**Fig. S24** a) P-XRD patterns, high-resolution XPS spectra of b) Co 2*p*, c) O 1*s*, and d) N 1*s* before the stability test and after 10 h of testing.

**Fig. S25** LSV curves of Fe_3_O_4_@MSA-Salen COFs with different sizes at 1600 rpm, recorded at a scan rate of 10 mV s^−1^ in 0.1 M KOH. a) Co-Salen COF and s/m-Fe_3_O_4_@Co-Salen COF, b) Fe-Salen COF and s/m-Fe_3_O_4_@Fe-Salen COF, c) Ni-Salen COF and s/m-Fe_3_O_4_@Ni-Salen COF.

**Fig. S26** electron transfer number of Fe_3_O_4_@MSA-Salen COFs with different sizes at 1600 rpm, recorded at a scan rate of 10 mV s^−1^ in 0.1 M KOH. a) Co-Salen COF and s/m-Fe_3_O_4_@Co-Salen COF, b) Fe-Salen COF and s/m-Fe_3_O_4_@Fe-Salen COF, c) Ni-Salen COF and s/m-Fe_3_O_4_@Ni-Salen COF.

**Fig. S27** a) LSV curves, b) electron transfer number, and c) H_2_O_2_ selectivity of Fe_3_O_4_ with different sizes at 1600 rpm, recorded at a scan rate of 10 mV s^−1^ in 0.1 M KOH. The chronoamperometry measurements at 0.2 V vs. RHE in 0.1 M KOH electrolyte of d) s-Fe_3_O_4_ and e) m-Fe_3_O_4_. f) The H_2_O_2_ yield and Faraday efficiency of s-Fe_3_O_4_ and m-Fe_3_O_4_.

**Fig. S28** LSV curves in Ar-saturated 0.1 M KOH containing 50 mM H_2_O_2_.

**Fig. S29** P-XRD patterns of s/m-Fe_3_O_4_@Co-Salen COF containing different amounts of s/m-Fe_3_O_4_.

**Fig. 30** a) LSV curves and b) electron transfer number of s-Fe_3_O_4_@Co-Salen COF containing different amounts of s-Fe_3_O_4_. c) LSV curves and d) electron transfer number of m-Fe_3_O_4_@Co-Salen COF containing different amounts of m-Fe_3_O_4_.

**Fig. S31** H-type electrolytic cell.

**Fig. S32** a) The calibration curve of K_2_TiO(C_2_O_4_)_2_ solution and b) the standard curve of its reaction with hydrogen peroxide.

**Fig. S33** The chronoamperometry measurements at varied applied voltages in a 0.1 M KOH electrolyte of MSA-Salen COFs.

**Fig. S34** The chronoamperometry measurements at varied applied voltages in a 0.1 M KOH electrolyte of s-Fe_3_O_4_@MSA-Salen COFs.

**Fig. S35** The H_2_O_2_ yield and Faraday efficiency of MSA-Salen COFs and s-Fe_3_O_4_@MSA-Salen COFs.

**Fig. S36** The H_2_O_2_ yield and Faraday efficiency of MSA-Salen COFs and s/m-Fe_3_O_4_@MSA-Salen COFs with different sizes.

**Fig. S37** Digital images for the decolorization of organic dyes and corresponding UV-vis spectra of RB.

**Fig. S38** In situ ATR-FTIR spectra of a) s-Fe_3_O_4_@Co-Salen COF and b) m-Fe_3_O_4_@Co-Salen COF catalysts in O_2_-saturated 0.1 M KOH solution (pH = 13) at different potentials from 0.8–0 V vs. RHE.

**Fig. S39** The DFT model of s-Fe_3_O_4_@Co-Salen COF and m-Fe_3_O_4_@Co-Salen COF.

**Fig. S40** Computed PDOS of Co-Salen COF, s-Fe_3_O_4_@Co-Salen COF, and m-Fe_3_O_4_@Co-Salen COF.

**Fig. S41** Computed PDOS of s-Fe_3_O_4_@Co-Salen COF and m-Fe_3_O_4_@Co-Salen COF after *OOH adsorption.

**S3 Supporting Tables**

**Table S1** ICP-OES results of MSA-Salen COF

| **Sample ID** | **Sample mass** *m*_0_ (g) | **Constant volume** *V*_0_ **(**mL) | **Element** | **Element concentration in test solution**  *C*_0_ **(**mg/L) | **Dilution factor f** | **Element concentration in original digestion solution**  *C*_1_ **(**mg/L) | **Element content in sample**  W **(**%) |
| --- | --- | --- | --- | --- | --- | --- | --- |
| Ni-Salen COFs | 0.0052 | 100 | Ni | 2.850 | 1 | 2.850 | 5.48% |
| Co-Salen COFs | 0.0046 | 100 | Co | 2.601 | 1 | 2.601 | 5.65% |
| Fe-Salen COFs | 0.0041 | 100 | Fe | 2.142 | 1 | 2.142 | 5.22% |

**Table S2** Curve fit parameters a for Zn-Salen COF

| **Sample** | **Shell** | ***CN*** | ***R*(Å)** | ***σ*^2^ (10^-3^ Å^2^)** | **Δ*E*_0_(eV)** | ***R*-factor** |
| --- | --- | --- | --- | --- | --- | --- |
| Zn-Salen COF | Zn-O | 2.3±0.03 | -0.01±0.05 | 0.005 | -7.72 | 0.007 |
|  | Zn-N | 2.0±0.01 | 0.09±0.03 | 0.007 | 6.43 |  |

Where *N* is the coordination number; *R* is the distance between absorber and backscatter atoms; *σ*^2^

is the *Debye*-*Waller* factor (a measure of thermal and static disorder in absorber-scatterer distances);

*R*-factor is used to value the goodness of the fitting.

**Table S3** The spin concentration of s-Fe_3_O_4_@MSA-Salen COF

| s-Fe_3_O_4_@MSA-SalenCOF | Zn | Fe | Co | Ni |
| --- | --- | --- | --- | --- |
| spin/g | 0 | 2.52×10^16^ | 2.24×10^16^ | 1.81×10^14^ |

**Table S4** Calculated band centers of the total Co *d*-electron and different orbitals

|  | Band center, eV | | | | |
| --- | --- | --- | --- | --- | --- |
| Co^2+^ status | *d*-total | *d*z_2_ | dxy | *d*xz-*d*yz | *dx*_2_-y_2_ |
| s-Fe_3_O_4_@Co-Salen COF | -2.67  -2.67 | -2.364 | -1.633 | -0.292  -0.292 | -1.821 |
| m-Fe_3_O_4_@Co-Salen COF | -2.26 | -2.143 | -2.021 | -0.009 | -2.284 |
| Co-Salen COF | -2.58 | -2.371 | -2.637 | 0.0337 | -2.80 |

**Supplementary References**

[S1] J.P. Perdew, K. Burke, M. Ernzerhof, Generalized gradient approximation made simple. Phys. Rev. Lett. **77**, 3865−3868 (1996). <http://doi.org/10.1103/PhysRevLett.77.3865>
[S2] A.G. Kresse, J. Furthmüller, Efficiency of ab-initio total energy calculations for metals and semiconductors using a plane-wave basis set-ScienceDirect. Comput. Mater. Sci. **6**, 15−50 (1996). <http://doi.org/10.1016/0927-0256(96)00008-0>
[S3] G. Kresse, J. Furthmüller, Efficient iterative schemes for ab initio total-energy calculations using a plane-wave basis set. Phys. Rev. B. **54**, 11169−11186 (1996). <http://doi.org/10.1103/physrevb.54.11169>
[S4] P.E. Blöchl, Projector augmented-wave method. Phys. Rev. B. **50**, 17953−17979 (1994). <http://doi.org/10.1103/PhysRevB.50.17953>
[S5] G. Kresse, D. Joubert, From ultrasoft pseudopotentials to the projector augmented-wave method, Phys. Rev. B. **59**, 1758−1775 (1999). <http://doi.org/10.1103/PhysRevB.59.1758>
[S6] S. Grimme, J. Antony, S. Ehrlich, H. Krieg, A consistent and accurate ab initio parametrization of density functional dispersion correction (DFT-D) for the 94 elements H-Pu. J. Chem. Phys. **132**, 154104 (2010). <http://doi.org/10.1063/1.3382344>
[S7] V. Wang, N. Xu, J.-C. Liu, G. Tang, W.-T. Geng, VASPKIT: A user-friendly interface facilitating high-throughput computing and analysis using VASP code. Comput. Phys. Commun. **267**, 108033 (2021). <http://doi.org/10.1016/j.cpc.2021.108033>
[S8] K. Momma, F. Izumi, VESTA 3 for three-dimensional visualization of crystal, volumetric and morphology data, J. Appl. Crystallogr. **44**, 1272−1276 (2011). <http://doi.org/10.1107/s0021889811038970>
